# Systemic failure and the totalization of public hospitals: a theoretical and conceptual synthesis of health governance in Latin America

**DOI:** 10.3389/fsoc.2026.1956466

**Published:** 2026-09-29

**Authors:** Mauro Marino Jiménez, Mariella Montes-Vecco, Nicole Nuñez-Zambrano

**Affiliations:** Facultad de Comunicación, Universidad San Ignacio de Loyola, Lima, Peru

**Keywords:** burnout syndrome, Latin America, public hospitals, social medicine, systemic failure, total institutions

## Abstract

The public hospital in Latin America is currently undergoing a sociological metamorphosis driven by chronic systemic failure. This article posits that, contrary to its design, the hospital frequently functions as a total institution by default—a space defined by surveillance, bureaucratic rationing, and the mortification of the self. Through a theoretical and conceptual synthesis grounded in a systematic documentary analysis of 31 primary units, this study contrasts the *external narrative* of performative governmental modernization with the internal reality of structural collapse. Integrating Goffman’s analytical heuristic with the framework of street-level bureaucracy, we analyze the “double erosion” of quality of life affecting both the workforce—manifesting in epidemic rates of burnout syndrome—and the patient population, who suffer systematic dehumanization. The findings reveal that staff depersonalization is a survival mechanism necessitated by resource scarcity, inadvertently causing the objectification of the patient. While the persistence of this crisis is maintained by a structural opacity that obscures the true diagnosis of the sector, counterexamples of primary care resilience demonstrate this totalization is not deterministic. We conclude that de-totalization requires not only meeting a GDP investment target but also a fundamental restructuring of the power dynamics within the clinical encounter.

## 1 Introduction: the total institution by default

Contrary to its teleological design, the public hospital in Latin America frequently functions as a total institution by default—a space increasingly characterized by surveillance, rigid control, and what Goffman (1998) termed the “mortification of the self”. This transformation represents an institutional adaptation to structural resource deficits, articulated across three intersecting axes of systemic strain.

At the structural-material level, an endemic deficit in healthcare financing—which consistently fails to meet the 6% GDP target recommended for universal health—creates an ecology of scarcity that necessitates the severe rationing of care (PAHO, 2025). This material void is compounded by organizational-bureaucratic challenges, where labor precarity and limited governance models can normalize moral injury among healthcare workers. In this high-pressure environment, procedural rigidity often emerges as a primary tool for managing overstretched demand, shifting the focus from therapeutic engagement to institutional containment (WHO, 2025; Segovia-Meza et al., 2025). Finally, these material realities are frequently obscured by discursive gaps, wherein a structural opacity maintains a fracture between the External Narrative of digital modernization and the *Internal Reality* of structural limitations (Da Silva Ferreira et al., 2024).

This totalization can be understood as the unintended consequence of prolonged institutional erosion. When a health system lacks the resources required for comprehensive care, it often pivots toward the bureaucratic management of patient populations through rationing and depersonalization (Munguía-Girón et al., 2021; IACHR, 2017). Consequently, the hospital risks functioning as an involuntary space of confinement, driven by systemic necessity rather than architectural intent.

The central thesis of this research is that this collapse culminates in a “Double Erosion” of quality of life, representing a simultaneous degradation of the subjective experience for both the provider and the recipient of care. Within this framework, the *totalization* of the hospital is mediated through a recursive cycle of trauma. Healthcare professionals are subjected to scarcity-induced pressures that manifest in epidemic rates of Burnout Syndrome, characterized specifically by a functional depersonalization (Munguía-Girón et al., 2021). To survive the psychological weight of chronic resource deficits, the staff must detach from their capacity for empathy as a defensive measure—a psychological shield that ensures professional survival at the cost of clinical humanity. As a result, this detachment triggers a secondary erosion for the patient, who undergoes a systematic stripping of dignity and identity. Within the environment of the total institution by default, the patient is reduced from a social subject with agency to a biological object to be managed, rationed, and processed (IACHR, 2017).

Therefore, this article asks: *How do chronic resource deficits and structural opacity narratives interact to theoretically transform the modern Latin American public hospital into a de facto total institution?* Through a theoretical and conceptual synthesis grounded in secondary literature, this study aims to deconstruct the dominant External Narrative and examine how systemic collapse mediates the clinical encounter.

## 2 Theoretical architecture: deconstructing the Goffmanian paradigm

To comprehend the depth of contemporary dehumanization, one must revisit and expand Erving Goffman’s (1998) foundational architecture of the total institution. Goffman defined these spaces as sites of residence and work where large numbers of like-situated individuals, sequestered from wider society, lead an enclosed and formally administered existence. However, applying this mid-century framework to the 2026 Latin American reality requires intersecting Goffman’s micro-sociology with the macro-sociological lenses of structural violence (Farmer, 2004) and biopolitics (Foucault, 2008). In this context, the hospital is not a “closed” building in the literal sense, but a closed system of logic where survival is contingent upon the submission of the self.

The institution asserts control through a process of systematic degradation that Goffman termed the “mortification of the self.” In the collapsed public hospital, this begins with role dispossession, where the patient is stripped of their extra-mural social identity—worker, parent, citizen—and reduced to a “hospital body” (Moglia and Sy, 2023). This is followed by a forced re-identification, where the subject is codified as a bed number, a diagnostic category, or a statistical burden to facilitate administrative processing over human engagement. Recent studies suggest this constitutes a form of clinical carcerality, where the architectural and bureaucratic barriers of the hospital function as a carceral sieve, filtering those *worthy* of care from those relegated to the waiting room (Castro and Erviti, 2015; IACHR, 2017).

In a functional medical model, the institution is teleologically oriented to serve the individual. In the collapsed public hospital, this relationship is inverted: the patient is placed at the disposal of the hospital’s operational limitations (Moglia and Sy, 2023). Patients are forced to navigate a “culture of waiting”—a temporal form of structural violence where the state exercises power by stealing the time of the marginalized (Auyero, 2012; Segovia-Meza et al., 2025).

This inversion represents a sophisticated biopolitical control. Here, the state manages the biological lives of its citizens not through the proactive provision of health, but through the calculated rationing of survival. The patient is no longer a citizen with a right to health, but a “unit of workload” to be managed, deferred, or neutralized. By transforming the clinical encounter into a site of surveillance and submissive compliance, the institution ensures its own survival at the expense of the ontological security of the patient (García-Pérez et al., 2025).

Crucially, the adaptation of Goffman’s “total institution” within this study operates as an analytical heuristic rather than a literal architectural equivalence (Goffman, 1998). While classic asylums maintained control through physical sequestering, the modern totalized hospital asserts control through temporal domination and bureaucratic rationing (Auyero, 2012; Moglia and Sy, 2023). This dynamic intersects Goffmanian totalization with Lipsky’s (1980) framework of street-level bureaucracy. In an ecology dictated by neoliberal managerialism—where formal efficiency metrics supersede quality of care—frontline healthcare workers function as street-level rationing agents who, facing unmanageable caseloads and chronic material deficits, develop defensive routines and depersonalized interactions to ration access and survive organizational strain (Lipsky, 1980; Segovia-Meza et al., 2025).

## 3 Analytical framework: contrasting narratives

This research employs a qualitative documentary analysis designed to examine the structural divergence within public health governance. As Bowen (2009) establishes, systematic document analysis enables the critical interpretation of institutional texts, policy briefs, and published empirical data to uncover latent organizational realities that official discourse obscures. By juxtaposing normative administrative reports against ground-level empirical ethnographies, this synthesis provides a triangulated evaluation of institutional transformation (Bowen, 2009; Biehl, 2005).

While this investigation methodologically adopts a qualitative documentary synthesis across 31 primary documentary units (Bowen, 2009), our interpretative synthesis is critically anchored in contextual, frontline observations within Peruvian public hospital triage settings during and following systemic health crises (Gianella, 2022; Segovia-Meza et al., 2025). Grounding the secondary corpus within situated authorial observations provides a necessary heuristic link between high-level macro-governance indicators and the micro-sociological reality of clinical rationing, triage negotiations, and supply procurement at the hospital bedside (Biehl, 2005; Moglia and Sy, 2023).

### 3.1 Discursive categorization: propaganda vs. precarity

The analysis is structured around the tension between two primary spheres of institutional communication:

The External Narrative (The Aesthetic of Modernization): This sphere encompasses the public-facing output of the state and health ministries. It is heavily characterized by an “indicator culture” (Gianella, 2022), where governments strategically deploy quantitative metrics and inaugurate “model hospitals” to project an image of efficiency (Giraudi and Pribble, 2020; Merry, 2016). This discourse is relentlessly future-oriented, emphasizing technological solutions to mask the foundational decay of the physical and human infrastructure.

The Internal Reality (The Phenomenology of Collapse): This sphere comprises data reflecting the actual socio-material operation of the system. It includes systematic reviews of staff morbidity—specifically epidemic rates of Burnout and moral injury—alongside technical reports regarding critical supply shortages, institutional shocks, and budgetary deficits (Castro et al., 2025). This narrative is present-oriented, defined by human suffering and an institutional “ecology of scarcity.”

### 3.2 Analytical logic: performative governance and structural opacity

The objective of this analytical heuristic is not to ascribe coordinated intentionality or conspiratorial coherence to political actors, but to analyze the sociological function of this discursive disconnect. In this study, the divergence between the External Narrative and the Internal Reality operates not as an intentional conspiracy, but as an emergent property of performative governance and structural opacity (McGoey, 2012; Da Silva Ferreira et al., 2024).

In clinical bioethics, the traditional conspiracy of silence or *pact of silence* describes the paternalistic withholding of terminal diagnoses to protect vulnerable patients from psychological harm (Da Silva Ferreira et al., 2024). Transposing this mechanism conceptually to macro-governance, institutional silence functions as an organizational defense mechanism rather than a malicious plot: the state apparatus and hospital administrations project operational stability (the paternalistic “provider”), while society absorbs unacknowledged systemic failure (the disempowered “patient”).

This dynamic produces what McGoey (2012) identifies as “strategic ignorance”—where institutions survive by institutionalizing blind spots, prioritizing audit metrics, and sanitizing systemic collapse to maintain operational legitimacy (Bourdieu, 1991; Gianella, 2022). Consequently, frontline personnel are caught in an administrative double-bind: they must validate official performance indicators on paper while managing acute material collapse in practice (Lipsky, 1980).

Furthermore, this analytical logic reveals the tragic role of the healthcare workforce within this structural opacity. The frontline staff are trapped in the discursive gap. To maintain their employment and institutional standing, they are forced to participate in the performativity of the External Narrative—filling out digital forms, adhering to superficial quality indicators, and rationing care quietly—while absorbing the trauma of the Internal Reality. By contrasting these two narratives, our analysis exposes how the total institution sustains its symbolic authority: it delegates the material consequences of state failure to the psychological endurance of its workers and the physiological suffering of its patients.

### 3.3 Data collection and corpus selection

The analytical framework relied on qualitative content analysis across 31 primary documentary units (Table 1). Textual data were extracted using a standardized coding instrument focused on two primary operational domains: (1) External Narrative Indicators, capturing state claims of digital modernization, quantitative metrics, and infrastructural investment; and (2) Internal Reality Indicators, capturing empirical data on workforce burnout, supply shortages, waiting times, and clinical dehumanization. Conceptually, findings were triangulated against Goffmanian indicators of totalization—specifically role dispossession, temporal domination, and linguistic erasure.

**Table 1:** Documentary units.

No.	Document citation	Geographic scope	Document/method type	Target analytical stream	Primary indicators/data extracted
01	PAHO (2025)	Pan-American	Macro-institutional report	External narrative/scarcity	GDP 6% expenditure target, financing shortfalls, systemic underfunding.
02	WHO (2025)	Global/LATAM	Global health monitoring report	Internal reality/scarcity	Health service disruptions, budget contraction, supply chain paralysis.
03	Katz (2023)	Regional (LATAM)	Socio-economic technical report	Internal reality/scarcity	Out-of-pocket health expenditures, informal privatization of care risk.
04	MINSA/DIGEMID (2024)	Peru	National Health surveillance & econometric report (ENAHO)	Internal reality/scarcity	Out-of-pocket spending on medicines (38.1–45.1%), retail pharmacy displacement (90.6%), and public insurance (SIS) financial vulnerability (45.7%).
05	Gianella (2022)	Peru	UNU-WIDER working paper	External narrative vs. internal	Indicator culture, performative governance, strategic ignorance in COVID-19.
06	Giovanella et al. (2018)	Regional (LATAM)	Policy & health systems analysis	External narrative	Slogans vs. material reality of Universal Health Coverage (UHC).
07	Giraudi and Pribble (2020)	Regional (LATAM)	Comparative political analysis	External narrative	Territorial inequality, centralization of high-tech “model hospitals.”
08	Batista (2021)	Regional (LATAM)	Global health policy brief	External narrative	Rhetorical framing of primary care vs. tertiary budget capture.
09	Hartmann (2019)	Nicaragua	Qualitative Policy Analysis	External Narrative	Discursive state rhetoric (“Vivir Bonito”) masking healthcare decay.
10	García-Pérez et al. (2025)	Regional (LATAM)	Systematic Review (2019–2023)	Internal reality (workforce)	Burnout prevalence (up to 70.3%), emotional exhaustion, staff depersonalization.
11	Munguía-Girón et al. (2021)	Regional (LATAM)	Systematic review (2015–2020)	Internal reality (workforce)	Baseline pre-pandemic burnout rates and depersonalization dynamics.
12	Castro et al. (2025)	LATAM & Caribbean	Epidemiological assessment	Internal reality (workforce)	Systemic shocks, facility paralysis, health workforce morbidity.
13	Coimbra et al. (2023)	Global/LATAM	Meta-analysis	Internal reality (workforce)	Moral injury among healthcare workers under chronic constraints.
14	Greenberg et al. (2020)	Global context	Clinical practice & mental health	Internal reality (workforce)	Moral distress etiologies caused by impossible clinical rationing.
15	Sale and Smith-Morris (2023)	Brazil	Qualitative bioethics study	Internal reality (workforce)	Structural violence and clinician coping mechanisms in public care.
16	Frias-Pérez and Acuña-Mayorga (2020)	Regional (LATAM)	Occupational health review	Internal reality (workforce)	Quality of life degradation among healthcare providers.
17	Takanohashi et al. (2025)	Regional (LATAM)	Econometric model	Structural context	Labor precarity, low wages, informal labor dynamics in public sectors.
18	Moglia and Sy (2023)	Regional (LATAM)	Meta-ethnography	Internal reality (patient)	Inversion of service relationship; patients re-labeled as “workload units.”
19	Auyero (2012)	Argentina	Ethnographic monograph	Internal reality (patient)	“Culture of waiting,” temporal violence, submission through delay.
20	Segovia-Meza et al. (2025)	Peru	Action-research & public health	Internal reality (patient)	Authoritarian procedural controls replacing empathetic clinical care.
21	Da Silva Ferreira et al. (2024)	Brazil	Bioethics & oncology study	Analytical logic	Performative governance, institutional defense mechanisms, and transposition of clinical pacts of silence to macro-governance.
22	Castro and Erviti (2015)	Mexico	Sociological monograph	Internal reality (patient)	Medical authoritarianism, obstetric violence, institutional discipline.
23	Castrillo (2020)	Argentina	Qualitative analysis	Internal reality (patient)	Dehumanization in public maternity wards and bodily autonomy loss.
24	Belli (2013)	Regional (LATAM)	Human rights report	Internal reality (patient)	Obstetric violence as normalized structural violation of dignity.
25	IACHR (2017)	Americas/Regional	Regional human rights report	Internal reality (patient)	Institutional gatekeeping, barriers to healthcare access, structural discrimination, and systemic degradation of marginalized patients.
26	Arbex (2013)	Brazil	Historical & investigative book	Case study 1 (extreme)	Hospital Colônia de Barbacena: 60,000 deaths, total mortification.
27	Coelho and Lindner (2021)	Brazil	Social sciences case study	Case study 1 (Extreme)	Necropolitics, role dispossession, and destruction of human dignity.
28	Herrera (2016)	Argentina	Field ethnography	Case study 2 (everyday)	Greater Buenos Aires maternity wards: linguistic erasure (“mamita”).
29	Horna-Saldaña and Perez (2025)	Peru	Case study investigation	Case study 3 (corrupt)	“Vacunagate”: vaccine diversion, elite bypass of institutional scarcity.
30	Mayta-Tristán and Aparco (2021)	Peru	Medical ethics analysis	Case study 3 (corrupt)	Misuse of trial vaccines and elite privilege in collapsed health systems.
31	Tagle et al. (2024)	Argentina & Peru	Media & political surveillance	Case study 3 (Corrupt)	Media exposure breaking the state’s structural opacity.

This table represents an inventory of the 31 primary documentary and empirical units utilized to inform the conceptual synthesis of this study. Purely theoretical foundational texts (e.g., Goffman, Foucault, Bourdieu) have been excluded from this index to strictly delineate the empirical boundaries of the documentary analysis. The corpus was systematically coded to contrast the External Narrative of macro-institutional governance against the Internal Reality of workforce morbidity and patient phenomenology across Latin American public health systems.

To ensure methodological reproducibility, the 31 documentary units were curated through a systematic search across major regional databases, including PubMed, Scopus, and SciELO. The search utilized boolean operators combining terms such as: *(“Burnout Syndrome” OR “occupational stress” OR “moral injury”) AND (“public hospital” OR “health system”) AND (“Latin America”)*.

The corpus boundaries were defined by strict inclusion and exclusion criteria. Included documents were limited to peer-reviewed empirical studies and official institutional reports published between 2012 and 2025, with a strict geographic focus on Latin American public healthcare contexts. Excluded from the corpus were studies focused exclusively on private healthcare networks, opinion pieces lacking verifiable data, and purely biomedical clinical trials lacking a sociological or public health governance dimension.

- Macro-Institutional and Discursive Data: To map the External Narrative and systemic baselines, we extracted data from high-level technical reports and institutional indicators. This included recent global monitoring reports from the World Health Organization (WHO) regarding service disruptions and the Pan American Health Organization’s (PAHO) frameworks on GDP investment targets.
- Sociological and Epidemiological Evidence: To document the Internal Reality, we synthesized findings from existing peer-reviewed literature focusing on the Latin American context. This involved compiling recent systematic reviews on the prevalence of Burnout Syndrome and occupational stress among regional healthcare workers, alongside foundational ethnographic studies detailing the “culture of waiting” and normalized obstetric violence.
- Qualitative Case Study Curation: The qualitative synthesis of institutional totalization relied on the targeted selection of historical, ethnographic, and media case studies. Specific data points and historical accounts regarding the *Hospital Colônia de Barbacena* in Brazil, routine obstetric violence in Argentina, and the *Vacunagate* clinical trial scandal in Peru were collected to provide phenomenological evidence of the theoretical model across extreme, every day, and corrupt dimensions.

## 4 The external narrative: performative governance and modernization

In the context of the total institution by default, the management of the external image is not merely a public relations exercise; it is a vital mechanism of institutional survival. Just as a carceral facility might invite inspectors to a model wing while obscuring solitary confinement, the Latin American health sector invests heavily in constructing a mirage of functionality. This External Narrative serves a dual purpose: it insulates the institution from political accountability and imposes a form of symbolic violence upon the public by denying the reality of their suffering (Bourdieu, 1991).

The official narrative functions primarily as performative governance designed to maintain political legitimacy. Health care is a primary battleground for the Latin American social contract; the ability to provide care is heavily scrutinized as a proxy for the state’s overall competence (Giovanella et al., 2018). Consequently, governments deploy large-scale conceptual plans as rhetorical shields. Slogans such as “Health for All” or “Universal Coverage” serve a performative function: they signal moral virtue without requiring the expenditure of political capital necessary to address the structural rot (Batista, 2021; Hartmann, 2019). These plans frequently emphasize community-based “Primary Care” in theory, while in practice, budgets remain captured by tertiary, curative institutions that are more visible and politically prestigious.

A potent motif in this External Narrative is the “*hospital modelo*” (model hospital). Ministries of Health frequently publicize the inauguration or high-tech renovation of specific facilities, presenting them as the standard for the entire system. Drawing on Baudrillard’s (1994) concept of the *simulacrum*, these hospitals function as hyper-real projections: they possess the aesthetic of functioning, modern hospitals—equipped with the latest imaging technology and immaculate corridors on the day of inauguration—but they serve to obscure the reality of the peripheral centers where the vast majority of the population seeks care (Giraudi and Pribble, 2020; Katz, 2023). By focusing public attention on the inauguration of a new wing, the administration deflects attention from the systemic absence of basic consumables like gauze, anesthetics, and antibiotics in the broader network.

Furthermore, the strategic use of digital management enables the quantification of “achievements” in a way that further perpetuates strategic ignorance (Gianella, 2022). Official reports highlight quantitative metrics: the number of patients processed, surgeries performed, or beds occupied. While literally accurate, these metrics are sociologically deceptive. What is omitted from the data is the quality and human cost of those encounters. A state report might celebrate “1,000 births attended,” deliberately omitting the normalized rates of obstetric violence, the rationing of anesthesia, and the grueling “culture of waiting” that accompanied them (Auyero, 2012; Castro and Erviti, 2015). This quantitative focus is a hallmark of bureaucratic rationality, which values the processing of bodies over the provision of care.

## 5 The internal reality: anatomy of a systemic collapse

Piercing the veil of the External Narrative, we encounter the *Internal Reality*—the documented, material reality of the hospital’s operation. This section dissects the socio-material conditions that transform the hospital into a total institution. The reality revealed here is not one of modernization, but of a desperate struggle for survival against institutional entropy.

### 5.1 The economic base: budgetary asphyxia and economic coercion

At the structural genesis of this totalization is an endemic investment deficit. The PAHO explicitly establishes that public expenditure on health of at least 6% of GDP is the foundational condition for universal coverage and quality care (PAHO, 2025). This is not an arbitrary metric; it is the calculated threshold for institutional sustainability. However, the majority of Latin American countries hover significantly below this threshold, creating a permanent, systemic “ecology of scarcity”.

The WHO (2025) recent global monitoring data underscores the operational impact of systemic underfunding, documenting extensive service paralysis across public facilities resulting from severe budget contractions (Castro et al., 2025; WHO, 2025). Within hospital wards, this macro-financial shortfall translates directly into chronic supply chain failures. Rather than an abstract budgetary deficit, regional empirical evaluations reveal widespread stockouts of basic clinical inputs—including sterile surgical gloves, disposable syringes, basic intravenous analgesics, pediatric antibiotics, suture thread, clean linens, and essential diagnostic reagents (Herrera, 2016; Moglia and Sy, 2023; Segovia-Meza et al., 2025).

In frontline triage and emergency rooms, this persistent material scarcity distorts the therapeutic mandate. Clinicians are routinely forced into an administrative calculus where therapeutic pathways are determined not by clinical evidence, but by the physical inventory of the cabinet during a given shift (Herrera, 2016; Moglia and Sy, 2023). When foundational consumables are depleted, practitioners face an ethical dilemma: suspend urgent procedures or hand the patient’s relatives a handwritten list to purchase items immediately from retail pharmacies operating outside the facility gates.

This dynamic drives an acute escalation of out-of-pocket health expenditure, effectively privatizing the economic risk of disease within nominally free public welfare networks (Katz, 2023; MINSA/DIGEMID, 2024). National surveillance data from Peru’s Ministry of Health confirms this systemic displacement: pharmaceutical purchases and basic consumables constitute the largest share of household out-of-pocket spending, accounting for 38.1% of all private health expenditures in 2023 (surpassing S/ 5,799 million) and reaching 45.1% among individuals with active health conditions. Crucially, 90.6% of this spending is displaced to commercial pharmacies and drugstores (*farmacias y boticas*) rather than being dispensed by public hospital pharmacies. Furthermore, patients enrolled in the non-contributory public system (SIS) absorb 45.7% of total national out-of-pocket health costs (MINSA/DIGEMID, 2024). This informal economic extraction operates as a secondary barrier of institutional exclusion: access to timely, dignified care becomes contingent upon immediate household liquidity, transforming public healthcare into a commodified, pay-to-access tier (IACHR, 2017; Katz, 2023; Segovia-Meza et al., 2025).

### 5.2 Organizational entropy and the vacuum of governance

Financial starvation is compounded by severe organizational entropy. The collapsed hospital functions not as a cohesive entity, but as a fragmented, feudal system of disconnected departments lacking centralized intelligence. The chronic scarcity of personnel—driven by layoffs, low wages, and labor precarity—leaves the remaining workforce dangerously overextended (Takanohashi et al., 2025).

Moreover, effective governance requires accurate data, yet the region suffers from an institutional *information blackout*. With interrupted surveillance and data collection systems (WHO, 2025), the health apparatus is effectively flying blind. Decisions are made not on epidemiological evidence, but on political intuition or crisis-management instincts. This deficient governance creates a vacuum of leadership. In the absence of coherent protocols and institutional backing, frontline staff are abandoned to manage the infinite demand of the public using finite resources. To survive this chaos, staff inevitably resort to authoritarianism and rigid procedural control, finalizing the hospital’s transformation into a total institution.

## 6 Structural entrapment: the epidemiology of professional exhaustion

In the classical sociology of the total institution, the staff are often viewed merely as the “oppressors” or the agents of surveillance. However, applying this framework to the collapsed Latin American hospital reveals a more complex and tragic dynamic. The frontline healthcare workers are, in fact, the “first inmates” of the collapsed system. They are trapped within the institution just as securely as the patients, bound by economic necessity, professional obligation, and the moral weight of their clinical mandate.

Recent systematic reviews mapping occupational stress in Latin American public healthcare from 2019 to 2023 document widespread but heterogeneous rates of psychological exhaustion (García-Pérez et al., 2025; Munguía-Girón et al., 2021). Burnout prevalence across public healthcare settings varies widely across professional cadres, specialties, and institutional environments, ranging from 13.2% up to observed maximums of 70.3% in high-strain sectors, characterized by severe emotional exhaustion (15.9–39.4%) and depersonalization (11.8–44.2%) (García-Pérez et al., 2025). Rather than representing a uniform, homogeneous structural fatality that erases individual agency, these figures illustrate the severe operational strain generated when public hospital capacity fails to meet clinical demand.

The etiologies of this strain are heavily linked to acute labor overload, staffing deficits, and diminished institutional backing (WHO, 2025; Frias-Pérez and Acuña-Mayorga, 2020). Under these conditions, the relationship between moral injury and burnout is best understood as complementary and sequential rather than competing: moral injury represents the foundational cognitive-ethical violation that occurs when clinicians are structurally prevented from delivering the care their professional ethics demand (Coimbra et al., 2023; Greenberg et al., 2020). Burnout Syndrome—specifically Maslach and Leiter’s (2016) dimension of depersonalization—develops as an adaptive psychological defense against this chronic ethical distress.

To illustrate this theoretical dynamic: consider an emergency room or triage setting where a single nurse faces an overcrowded waiting room exceeding physical capacity. In such high-stress scenarios documented across regional ethnographies (Moglia and Sy, 2023), emotional detachment becomes an operational survival shield. To prevent psychological collapse, workers may subconsciously objectify patient populations—treating incoming individuals as clinical tasks or bed numbers rather than suffering persons (Maslach and Leiter, 2016; Sale and Smith-Morris, 2023).

This depersonalization directly catalyzes the dehumanization of the patient. If clinicians allow themselves to internalize the full scope of structural suffering within an overcrowded ward, emotional exhaustion becomes inevitable. Empathy becomes an operational liability in an ecology of scarcity. Thus, staff enforce rigid rules and maintain clinical distance not out of inherent malice, but as an institutional survival strategy (Sale and Smith-Morris, 2023). The frontline worker becomes an instrument of totalization precisely because they are compromised by systemic neglect.

## 7 Institutional mortification: the phenomenology of the patient experience

The terminal result of budgetary asphyxia and staff depersonalization is the “mortification of the self” of the patient (Goffman, 1998). This section details the phenomenology of being a patient in a total institution by default, describing the lived experience of the citizen who enters the public hospital seeking sanctuary and instead encounters a machinery of control.

### 7.1 Identity erasure, gendered relabeling, and ontological reduction

The process of institutional mortification begins with the dispossession of individual identity (Goffman, 1998). Ethnographic and observational studies in regional public maternity and emergency wards reveal that staff frequently abandon the use of patients’ proper names (Castro and Erviti, 2015; Herrera, 2016; Moglia and Sy, 2023). Instead, patients are relabeled through colloquial diminutives such as “*mamita*” (little mother) or derogatory descriptors like “*gorda*” (fatty), or reduced to spatial markers such as “la de la cama 2*”* (the one in bed 2).

Critically, this linguistic erasure exhibits a pronounced gender asymmetry. The relabeling of female patients as *“mamita”* is not merely an informal colloquialism; it is a manifestation of institutional symbolic violence (Bourdieu, 1991; Belli, 2013). By reducing adult women exclusively to their reproductive capacity and infantilizing them through diminutives, the medical encounter strips them of clinical autonomy, leaving little room for informed dissent or collaborative care (Castrillo, 2020; Herrera, 2016). This dynamic reflects broader gender-based power structures across Latin American welfare sectors, where women’s physical bodies and reproductive choices are disproportionately subjected to patriarchal institutional surveillance and state moralizing (Castro and Erviti, 2015; UN Women, 2023). Concurrently, this loss of naming facilitates clinical detachment: when personal identity is replaced by a biological archetype, patient vulnerability is more easily managed within an overextended bureaucratic system.

### 7.2 Temporal domination and the inversion of service

Beyond spatial and bodily control, one of the most potent mechanisms of totalization is the manipulation of time. Ethnographies of the clinical encounter describe a pervasive “culture of waiting” (Auyero, 2012), where the marginalized patient’s time is treated as inherently valueless. Patients wait hours for a triage consultation, days for an available bed, and months for surgical interventions.

This temporal delay is not just an administrative bottleneck; it is a display of biopolitical power. Flexibility emerges as a privilege of status: the institution operates on its own opaque schedule, while the patient must remain permanently available, placed completely at the disposal of the hospital (Moglia and Sy, 2023). They cannot leave to secure food or care for dependents lest they lose their precarious place in line. This enforces a learned passivity, teaching the patient their precise location in the structural hierarchy: they are supplicants, and their time is forfeit to the state.

To further manage this scarcity, staff often resort to informal moral categorization to determine who receives attention. The “good” patient is defined by their submissiveness—they accept the wait and the lack of resources with quiet gratitude. Conversely, the “bad” patient is one who demands information or protests the delay. Ethnographic data indicates that staff frequently employ class-based stereotypes to label demanding patients from lower socioeconomic backgrounds as morally deviant or dangerous (Moglia and Sy, 2023). By framing the patient as a threat or a nuisance, the staff construct an “alibi” that justifies the harshness of their treatment, thereby cementing the inversion of the therapeutic relationship into a carceral one.

## 8 Case studies in institutional totalization

While Latin America is characterized by significant heterogeneity in health financing and welfare regimes, the phenomenon of totalization emerges predominantly in systems suffering from acute structural neglect. To illustrate the phenomenological spectrum of this totalization—and to acknowledge pathways of institutional resilience—we present four distinct case studies.

### 8.1 Case 1: the extreme - the *Holocausto Brasileiro* (Brazil)

The *Hospital Colônia de Barbacena* represents the historical limit-case of the total institution in the region. While it was systematically dismantled during the psychiatric reforms of the late 20th century, its shadow persists as a testament to the necropolitical endpoint of institutional logic. Functioning for decades, this psychiatric hospital devolved into a carceral dumping ground for the socially undesirable. Admission criteria were rarely medical; rather, they were mechanisms of social cleansing. The institution housed the marginalized: political dissidents, LGBTQIA+ individuals, unwed mothers, the extreme poor, and the disabled (Arbex, 2013; Coelho and Lindner, 2021).

Barbacena functioned not as a site of healing, but as an apparatus for the physical and social elimination of the “unproductive.” The outcome was catastrophic: an estimated 60,000 people died within its walls from starvation, exposure, and disease. The inmates were stripped of all ontological status, forced into slave labor, and subjected to absolute mortification. The dehumanization was so absolute that, upon death, bodies were routinely sold to medical universities for anatomical study. The patient was reduced to an object in life and a commodity in death. Barbacena represents the *genocide* that occurs when structural, ethical, and organizational failures remain unchecked by the state (Arbex, 2013).

### 8.2 Case 2: the everyday - obstetric violence (Argentina)

If Barbacena is the historical exception, the modern obstetric center in Greater Buenos Aires represents the rule. It exemplifies the “banality” of the total institution—the grinding, daily humiliation that is normalized within the clinical encounter. In standard public maternity wards, ethnographic and sociological studies document the routine mechanisms of dehumanization embedded in the hospital workflow (Belli, 2013; Castrillo, 2020).

Here, the mortification of the self is subtle but pervasive. Laboring women are systematically denied information regarding their clinical progress, leaving them in a state of manufactured uncertainty. They are frequently subjected to moralizing discipline by burnt-out staff (“If you liked making the baby, don’t complain now”), which punishes the expression of suffering and enforces submissiveness. The linguistic erasure is absolute, with women referred to by infantilizing nicknames rather than their names. This violence is invisible precisely because it is routine; it is justified internally as “hospital protocol” necessitated by a “scarcity of staff.” It demonstrates how the inversion of the service relationship functions to manage patient flow at the direct expense of bodily autonomy and dignity (Castrillo, 2020).

### 8.3 Case 3: the corruption - *Vacunagate* (Peru)

The 2021 *Vacunagate* scandal illustrates how the total institution is also a site of heavily stratified privilege, revealing that the “walls” of institutional scarcity are highly permeable for the elite. During the deadliest wave of the COVID-19 pandemic, while the Peruvian public hospital system completely collapsed—leaving citizens to die in waiting rooms or on the streets due to a lack of oxygen—hundreds of doses of the Sinopharm vaccine intended for clinical trial staff were secretly diverted. The recipients were the nation’s political, business, and medical elites, including the sitting president and the lead researchers’ families (Horna-Saldaña and Perez, 2025; Mayta-Tristán and Aparco, 2021; Tagle et al., 2024).

This case reveals a critical sociological caveat: the rules of the total institution (rationing, waiting, depersonalization) apply strictly to the marginalized ‘inmates’ (the general public). The elite possess a clandestine backdoor. This permeability demonstrates that the systemic failures described in this paper are not merely the result of administrative incompetence, but of a structural inequality that values certain biological lives over others. It shattered the façade of equal scarcity, proving that the state possessed the capacity to secure life-saving resources, but selectively reserved them for those with political capital (Gianella, 2022; Mayta-Tristán and Aparco, 2021).

The cases presented in Brazil, Argentina, and Peru are exemplary and illustrate recurring situations in the region (Table 2).

**Table 2:** Manifestations of the total institution in Latin American health systems.

Case manifestation	Location	Context	Key sociological mechanism	Consequence/impact
The extreme (*Hospital Colônia de Barbacena*)	Brazil	Psychiatric hospital functioning as a site for social confinement.	Absolute role dispossession and necropolitics; patients treated as objects in life and commodities in death.	Approximately 60,000 deaths; the ultimate consequence of unchecked ethical and organizational failure.
The everyday (obstetric violence)	Argentina	Standard public maternity wards.	Routine mortification of the self; linguistic erasure, denial of information, and moralizing discipline.	Normalization of systemic violence; the patient endures an inversion of service to manage hospital workflow.
The corruption (“vacunagate”)	Peru	Clinical trials for COVID-19 vaccines.	Permeability of the total institution’s rules for elites; circumvention of institutional scarcity.	Shattered public trust; reinforces that systemic rationing and waiting apply exclusively to vulnerable populations.

The qualitative synthesis of institutional totalization is based on historical, ethnographic, and media case studies regarding the Hospital Colônia de Barbacena in Brazil (Coelho and Lindner, 2021), obstetric violence in Argentina (Herrera, 2016), and the “Vacunagate” scandal in Peru (Tagle et al., 2024; Horna-Saldaña and Perez, 2025).

### 8.4 Case 4: the resilience - primary care and De-totalization (Costa Rica)

Situating Costa Rica’s primary care success within the contemporary regional landscape reveals an important contrast against broader continental political realignments. Over recent political cycles, several Latin American nations—including Argentina, Peru, and neighboring administrations—have experienced political shifts toward conservative and austerity-oriented governance frameworks. In many of these contexts, public health restructuring has been characterized by fiscal retrenchment, deregulation, and the reduction of state-funded social welfare umbrellas, further exacerbating the material precarity of public hospitals (Giraudi and Pribble, 2020; Katz, 2023).

In contrast to this continental trend of budgetary containment, Costa Rica’s model illustrates the protective role of institutionalized social pacts. By sustaining a public health investment exceeding 7% of GDP and insulating its primary care infrastructure (EBAIS) from austerity cycles, Costa Rica maintains public healthcare as an integrated social right rather than a residual safety net. This comparison confirms that hospital totalization is neither an intrinsic cultural feature of Latin American public administration nor an inescapable destiny; rather, it is directly conditioned by macro-political governance paradigms and the willingness of the state to insulate healthcare financing from ideological cycles of austerity.

## 9 Conclusions and pathways to de-totalization

Moving beyond the purely descriptive application of Goffmanian heuristics, this theoretical synthesis demonstrates that the totalization of Latin American public hospitals is not an inevitable cultural condition or an intentional architectural design, but an emergent operational pathology driven by chronic systemic underfunding and structural opacity. When public health networks are systematically starved of material resources, institutional containment, temporal queuing, and defensive depersonalization emerge as default survival strategies.

Diagnosing the public hospital as a *total institution by default* shifts the analytical and policy focus: worker cynicism and patient mortification can no longer be dismissed as individual moral failings or resolved through superficial “resilience training.” They must be understood as systemic adaptations to an impossible clinical mandate.

A critical finding of this synthesis is that institutional totalization is not politically deterministic. As demonstrated by the counterexample of Costa Rica, sustained primary-care infrastructure and health financing above 7% of GDP can successfully buffer clinical encounters, protect patient dignity, and disrupt the cycle of depersonalization. However, the Costa Rican case must be understood against the broader continental landscape. Across Latin America—in nations such as Argentina, Peru, and neighboring contexts—recent political realignments toward conservative and fiscal-austerity models have accelerated the retrenchment of public healthcare investment. In many settings, this shift has compounded labor precarity, centralized prestige budgets in tertiary facilities, and transferred the economic burden of care onto marginalized households through informal out-of-pocket costs. The divergence between these trajectories confirms that hospital totalization is directly mediated by macro-political choices: whether a state treats health as a universal social right or reduces it to a residual, rationed safety net.

To interrupt this cycle and initiate actionable processes of De-Totalization, public health governance must move beyond performative modernization and enact three structural transformations:

- Decoupling Allocation from Tertiary Prestige (The Economic Shift): Meeting the PAHO baseline of 6% of GDP dedicated to public health is an urgent material floor, not an optional aspirational target. Crucially, fiscal allocations must be actively rebalanced away from high-visibility, politically prestigious “model hospitals” and channeled directly into primary healthcare (PHC) networks. Strengthening territorial primary care intercepts pathology before it reaches tertiary centers, preventing the systemic saturation that forces hospitals into custodial containment. Simultaneously, public financing must guarantee the supply chain of basic consumables to eliminate the predatory out-of-pocket expenditures that currently require patients to subsidize state failure.
- Institutionalizing Data Transparency to Dismantle Opacity (The Organizational Shift): Reversing performative governance requires dismantling institutional opacity. Health ministries must transition away from sanitized aggregate indicators (e.g., raw throughput of attended cases) that conceal clinical degradation. In their place, administrations must institutionalize open, real-time public reporting on supply stocks, diagnostic backlogs, and patient wait times. Transparent data production neutralizes the bureaucratic impulse to hide systemic collapse behind digital veneers, creating genuine accountability for institutional resource deficits.
- Labor Restructuring to Remediate Moral Injury (The Human Strategy): Clinical humanization cannot be extracted from an ethically traumatized and materially abandoned workforce. Health governance must formally recognize moral injury—the distress caused by being forced to violate professional ethical commitments due to institutional scarcity—as the primary structural etiology of clinical burnout. Reversing provider detachment requires enforceable safe-staffing ratios, labor formalization, and institutional protocols that remove individual clinicians from the role of unbuffered rationing agents. Only by restoring the occupational security of healthcare providers can the therapeutic encounter be reclaimed as a collaborative partnership rather than an adversarial dynamic of surveillance and submission.

The public hospital does not have to function as a carceral sieve. Reclaiming its teleological purpose requires piercing the veil of performative modernization, directly funding the material base of clinical care, and dismantling the structural hierarchies that transform vulnerable citizens into mere units of bureaucratic processing.

### 9.1 Limitations and reflexivity

This theoretical synthesis is not without limitations. By relying primarily on a documentary corpus of macro-institutional reports and existing meta-ethnographies, this analysis operates at a macro-sociological level. It may consequently overlook micro-level clinical variations, individual acts of profound empathy, and localized pockets of resilience that persist within these collapsed systems. Furthermore, as researchers situated within the Peruvian academic and clinical sphere, our institutional exposure to public hospital triage dynamics informs our interpretation of bureaucratic rationing and material exhaustion. Having observed frontline clinical encounters where staff are compelled to negotiate supply purchases with patients’ families prior to treatment, our focus on structural violence reflects real operational constraints. Future empirical research should expand on these findings through multi-sited, comparative hospital ethnographies that document micro-level variations in how clinical wards resist or succumb to supply precarity.
